# A comprehensive scoring system for defecation disorders derived by merging various validated patient-reported outcome measures for fecal incontinence, chronic constipation, and obstructed defecation

**DOI:** 10.1007/s00384-026-05086-x

**Published:** 2026-01-29

**Authors:** Carlo Ratto, Ilaria Simonelli, Paola Campennì, Francesco Litta, Mario Pagano, Angelo Parello, Angelo Alessandro Marra

**Affiliations:** 1 Proctology and Pelvic Floor Surgery Unit, Center of Excellence for Gastrointestinal and Endocrine-Metabolic Diseases, Isola Tiberina—Gemelli Isola Hospital, Via di Ponte Quattro Capi, 39, 00186 Rome, Italy; 2 Department of Medical and Surgical Sciences, Università Cattolica del Sacro Cuore, Rome, Italy; 3 Biostatistical Service, Clinical Trial Center, Isola Tiberina–Gemelli Isola Hospital, Rome, Italy; 4 Department of Biomedicine and Prevention, University of Rome Tor Vergata, Rome, Italy

**Keywords:** Patient-reported outcome measure, Defecation disorders, Fecal incontinence, Chronic constipation, Obstructed defecation syndrome

## Abstract

**Purpose:**

Currently, too many Patient-Reported Outcome Measures (PROMs) with redundant and repetitive domains are adopted to assess defecation disorders, resulting in more extended clinical visits and increased patient burden. The aim of this study was to develop a new comprehensive Defecation Disorders Scoring System (DDSS) by incorporating all items of the most commonly used and validated PROMs.

**Methods:**

This is a prospective observational study on patients waiting for rectal prolapse and defecation disorders surgery. Preoperatively, each patient completed seven different authoritative PROMs, two questionnaires assessing constipation, two questionnaires for obstructed defecation, two questionnaires to evaluate fecal incontinence, and one questionnaire aiming to assess both. Spearman’s correlation and Principal Component Analysis with varimax rotation were applied. Internal consistency was evaluated using Cronbach’s *α*.

**Results:**

A total of 127 female patients completed all 57 items across the seven validated PROMs and were included. Several items highly correlated with others expressing the same concept were reconsidered and excluded. A final set of 19 items was identified and arranged into DDSS, encompassing five core components regarding specific aspects of incontinence, bowel movements/defecation frequency, evacuation effort and duration, type of assistance, and abdominal discomfort. Regarding internal consistency, the derived DDSS and its five components demonstrated satisfactory results.

**Conclusions:**

This study highlights the potential for reducing item redundancy across existing PROMs for defecation disorders. Despite some limitations, the proposed DDSS could potentially provide a concise, comprehensive tool for assessing multiple aspects of defecation disorders, potentially available in electronic format. Future studies will be required to further evaluate and validate DDSS across different patient populations.

## Introduction

The term Patient-Reported Outcome Measure (PROM) is defined as any report on a patient’s health status provided directly by the patient, without interpretation by healthcare professionals [1–3]. These multi-item questionnaires gather information on various aspects of a patient’s condition or lifestyle, providing a quantification of the patient’s perception of symptom severity and its impact on daily life through a standardized approach [1–4]. They can be used in both clinical and research settings as primary or secondary outcome measures when assessing treatment efficacy or impact [4]. Since the success of a course or treatment can no longer be determined by a single outcome measure alone, clinicians should also consider the patient’s ability to proceed through life in a functional and satisfactory manner [5]. Additionally, the routine use of validated PROMs ensures that all necessary questions are asked at every stage.

Several questionnaires have been developed to assess bowel, bladder, sexual, and pelvic organ prolapse dysfunctions and their impact on patient’s quality of life (QoL) [6]. Furthermore, the complexity and heterogeneity of these disorders highlight the need for a more comprehensive assessment strategy that integrates both clinical aspects and PROMs to better understand the pathophysiology of each condition and its subsequent effect on patients’ perceptions, especially before and after treatment [5, 6]. Defecation disorders (DDs), including fecal incontinence (FI), chronic constipation (CC), and obstructed defecation syndrome (ODS), are such complex and distressing conditions that PROMs are essential for thoroughly understanding their impact on a patient’s life and providing effective treatment [7, 8]. Moreover, different DDs often coexist, making the scenario more challenging [9]. In fact, too many PROMs have been developed and validated for DDs [6, 7, 10, 11]. However, no single PROM can offer a comprehensive interpretation of all DD aspects, leading to multiple PROMs being filled out by patients, which increases the time required and results in redundant or repetitive information. Therefore, this study aimed to propose a new DDs Scoring System (DDSS) by incorporating items from the most widely used and validated PROMs for DDs, with the intention of providing a comprehensive assessment tool.

## Methods

A prospective, single-center observational study was conducted on a consecutive cohort of female patients suffering from rectal prolapse (RP) and DDs, including FI, CC, and ODS, observed at our tertiary academic center from November 2020 to November 2024. Patients with RP were selected for their potential to report different DD aspects, which frequently coexist. In particular, female patients were predominantly selected due to the higher prevalence of RP in this population and to ensure a more homogeneous sample, given that only a small number of male patients underwent surgery for RP at our Unit during the study period. Exclusion criteria were males, malignancy under treatment, inflammatory bowel disease, refractory chronic diarrhea, neurologic disorders, pregnancy, severe comorbidities, and inability to give written informed consent. The study was approved by our Local Ethical Committee (ID 6120), and written consent was obtained from all participants. The STROBE statement for cohort studies was adopted [12].

### Study design

Each patient completed several validated PROMs assessing different DD aspects before surgery:

- two questionnaires assessing CC (Cleveland Clinic Constipation Scoring System—CCSS [13] and Patient Assessment of Constipation Symptoms—PAC-SYM [14]);
- two questionnaires for ODS (Altomare ODS score [15] and Longo ODS score [16]);
- two questionnaires regarding FI (Cleveland Clinic Fecal Incontinence Score—CCFIS [17] and St. Mark’s Incontinence Score—SMIS [18]);
- one questionnaire aiming to assess both CC, ODS, and FI, although certain aspects are not comprehensively addressed and are only marginally discussed (Symptom Severity Score—SSS [19]).
- Patients filled in all PROMs independently. When questions caused uncertainty, clinicians provided simplified explanations without influencing responses.

#### Questionnaire details

The CCSS is an 8-item scoring system designed to evaluate CC severity [13]. It has been adopted and validated worldwide and is especially valued for its user-friendly simplicity. The PAC-SYM is a questionnaire for patients suffering from CC [14]. It is one of the most well-developed and validated PROMs for CC.

The Altomare ODS score is an 8-item questionnaire designed to assess patients with ODS [15]. Although its responsiveness was not specifically tested, the Altomare ODS score’s discriminant validity was confirmed, and it is widely used in evaluating treatments for ODS. The Longo ODS score is commonly used to assess the effectiveness of surgery after the Stapled Transanal Rectal Resection (STARR) procedure for ODS [16]. The SSS is a 9-item PROM designed to evaluate CC, ODS, and FI [19]. Although the SSS and Longo ODS scores have not been formally validated, they were commonly and widely employed to assess surgical efficacy after STARR surgery in large surgical series [16, 19].

The CCFIS was introduced by Wexner and Jorge in 1993 to evaluate FI aspects, but its reliability, validity, and responsiveness were later confirmed by several other studies [11, 17]. The SMIS was derived from the CCFIS by simply adding two new items—one assessing urgency and one regarding the use of constipating medication—and modifying frequency descriptors for different types of FI (changing from “usually” to “weekly,” and from “always” to “daily”) [18].

### Statistical analysis

Principal component analysis (PCA) with varimax rotation was applied to reduce the number of items. PCA is a dimensionality reduction technique that helps remove redundant items by creating composite scores (components) representing clinical domains that capture the maximum variance while preserving the most essential information. Before performing PCA, two adequacy tests—the Bartlett test of sphericity and the Kaiser–Meyer–Olkin (KMO) test—were conducted. KMO values range from 0 to 1, and a value above 0.6 indicates the sample is suitable for analysis [20, 21]. The principal component extraction was based on the Kaiser Criterion (keeping components with eigenvalues greater than 1). Items were removed if they had low communality or cross-loaded onto more than one component. An item that loaded at 0.32 or higher on two or more components was considered a cross-loading item [22].

Spearman’s correlation coefficient was also calculated and, to avoid redundancy, one item from each pair of highly correlated items measuring similar concepts was automatically retained. Finally, statistical analysis using Cronbach’s *α* was performed to assess DDSS’s internal consistency. Questionnaires with any missing data or unanswered items were excluded from the statistical analysis. A *p*-value < 0.05 was considered statistically significant. Statistical analyses were conducted using IBM SPSS Statistics for Windows, Version 26.0 (IBM Corp., Armonk, New York, USA), and Python in Excel (Anaconda Inc., Austin, Texas, USA).

## Results

**Fig. 1:**
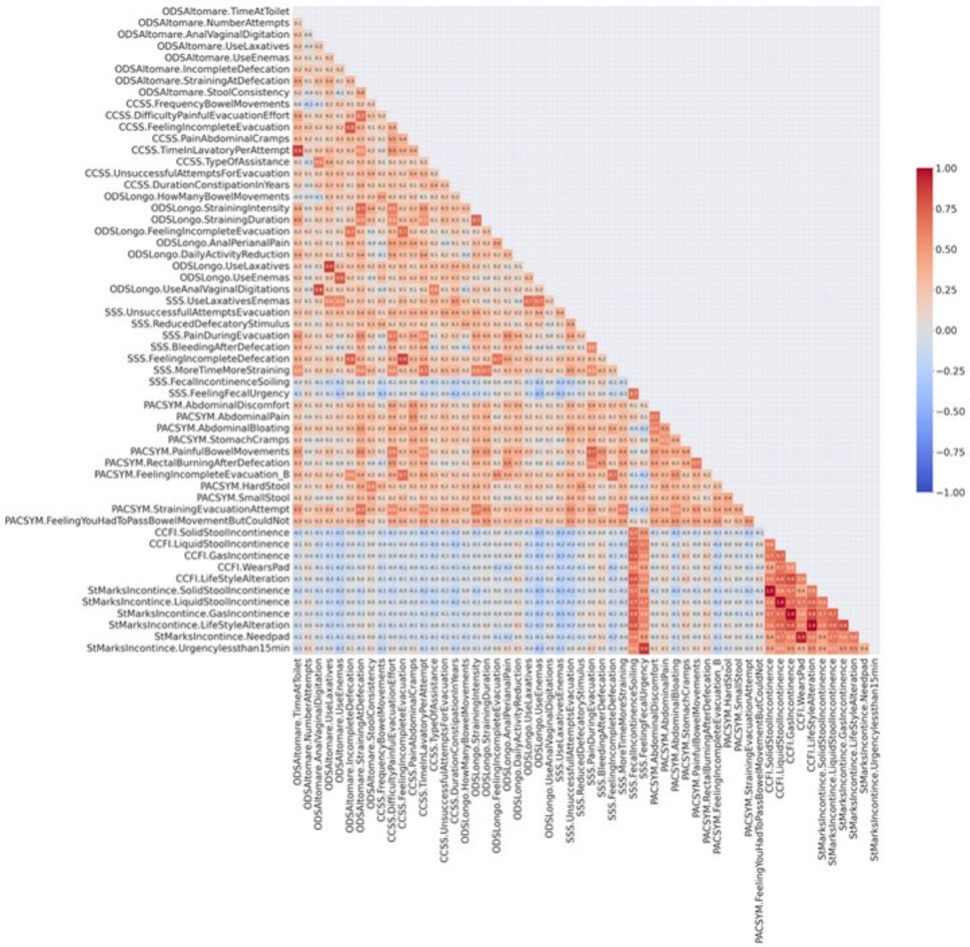
Correlation map among all 57 items of the seven selected Patient-Reported Outcome Measures with each other. The more the color tends toward red, the stronger the correlation between the two items. ODS, obstructed defecation syndrome; CCSS, Cleveland Clinic Constipation Scoring System; SSS, Symptom Severity Score; PAC-SYM, Patient Assessment of Constipation Symptoms; CCFI, Cleveland Clinic Fecal Incontinence Score

During the study period, 127 out of 159 (79.9%) female patients with RP completed all seven validated PROMs, comprising a total of 57 items related to FI, CC, and ODS. Before performing PCA, the item on daily defecation attempts in the Altomare ODS score was removed due to its low correlation with the others (rho coefficients ranged from −0.18 to 0.27). The item concerning the assumption of constipating medication in the SMIS was not considered, as it addressed a specific aspect that was opposite to the other CC and ODS factors. Examining the correlation matrix (Fig. 1), any items that were highly correlated with others or that expressed the same concept in a similar way were also excluded.

More specifically:

- the item on mean time spent on the toilet in the Altomare ODS score was removed due to high correlation with the time spent in the lavatory per attempt in the CCSS (*r* = 0.87);
- the use of anal/vaginal digitations in the Longo ODS score was excluded because it conveyed the same concept as the item on anal/vaginal digitations in the Altomare ODS score (*r* = 0.84);
- the item on laxatives use in the Longo ODS score was highly correlated with laxatives use in the Altomare ODS score (*r* = 0.88) and laxatives/enemas use in the SSS (*r* = 0.70), so only the first item was retained;
- the item regarding enemas/suppositories in the Altomare ODS score was removed because it expressed the same concept as in the Longo ODS score (*r* = 0.77);
- the feeling of incomplete evacuation in the CCSS was highly correlated with items measuring that feeling in the SSS (*r* = 0.85), PAC-SYM (*r* = 0.70), Altomare (*r* = 0.78), and Longo (*r* = 0.70) ODS scores, so only the first was chosen;
- the intensity of straining in the Longo ODS score overlapped with items on straining during defecation in the Altomare ODS score (*r* = 0.72) and straining during defecation attempts in the PAC-SYM (*r* = 0.70), so only the first one was retained;
- the item on pain during defecation in the PAC-SYM was removed because it duplicated the concept of pain on opening bowels in the SSS (*r* = 0.74);
- the question on soiling frequency in the SSS was highly correlated with the frequency of liquid stool leakage in the CCFIS (*r* = 0.74) and SMIS (*r* = 0.74), so only the first item was retained;
- the frequency of lifestyle changes related to FI in the SMIS was excluded because it was perfectly correlated with the item on the frequency of lifestyle changes for FI in the CCFIS (*r* = 1);
- the question about the need for a pad frequency for FI in the SMIS was removed because it conveyed the same concept as the item on pad frequency in the CCFIS (*r* = 0.95).

PCA was then applied iteratively, with cross-loading items removed after each run to establish a strong structure, as summarized in Table 1.

**Table 1:**
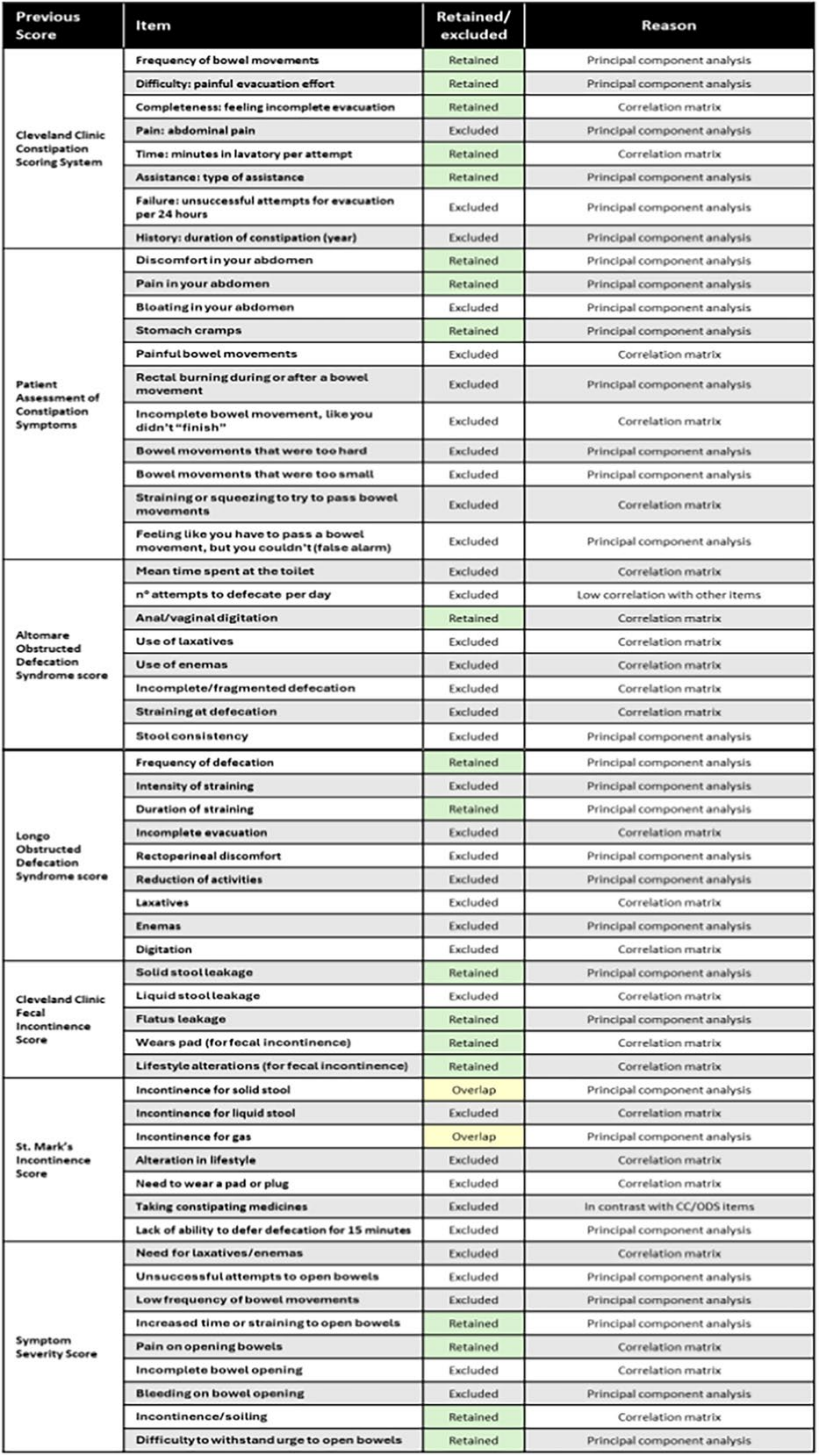
All items from the seven selected Patient-Reported Outcome Measures on defecation disorders, indicating which items were retained or excluded, with particular emphasis on the reasons for their inclusion/exclusion (specifically detailing the statistical methods applied). *CC *chronic constipation, *ODS* obstructed defecation syndrome

Green indicates items retained in the statistical process; yellow indicates items retained but overlapping between the Cleveland Clinic Fecal Incontinence Score and the St. Mark's Incontinence Score

**Fig. 2:**
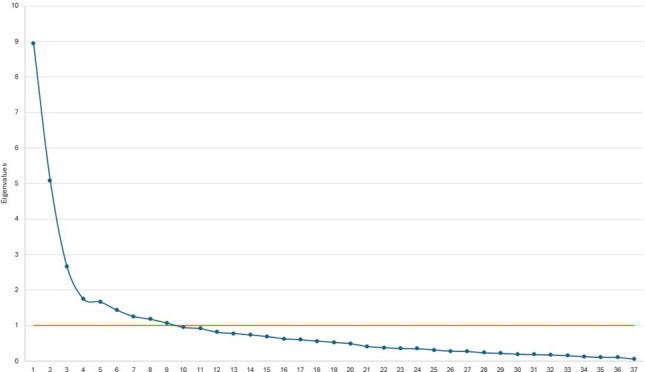
Eigenvalue Scree Plot shows the nine components selected according to the Kaiser criterion, accounting for 67.9% of the total variance

The Bartlett’s test of sphericity was significant (*p* < 0.001), confirming suitability for PCA, and the Kaiser–Meyer–Olkin (KMO) measure of 0.82 indicated sampling adequacy. Initially, nine components were selected following the Kaiser Criterion (with the first components having significantly higher eigenvalues), as shown in the eigenvalue scree plot (Fig. 2). These nine components accounted for 67.9% of the total variance.

After five iterations, a robust five-component model explaining 68.9% of the variance was achieved. Based on the loading matrix, the following results were observed:

- Component 1 exhibited high loadings for items addressing incontinence (SSS—fecal incontinence/soiling, difficulty to withstand urge to open bowels; CCFIS/SMIS—solid stool incontinence, gas incontinence, wears pad, lifestyle alteration);
- Component 2 showed high loadings for items related to the frequency of bowel movements/defecation (CCSS—frequency of bowel movements; Longo ODS score—frequency of defecation);
- Component 3 showed high loadings for items on evacuation effort and duration (CCSS—time: minutes in lavatory per attempt, completeness: feeling incomplete evacuation, difficulty: painful evacuation effort; SSS—pain on opening bowels, increased time or straining to open bowels; Longo ODS score—duration of straining);
- Component 4 showed high loadings for items addressing the type of assistance (CCSS—assistance: type of assistance; Altomare ODS score—anal/vaginal digitation);
- Component 5 showed high loadings for items related to abdominal discomfort (PAC-SYM—discomfort in your abdomen, pain in your abdomen, stomach cramps).

The final developed questionnaire included 19 items listed in Table 2.

**Table 2:**
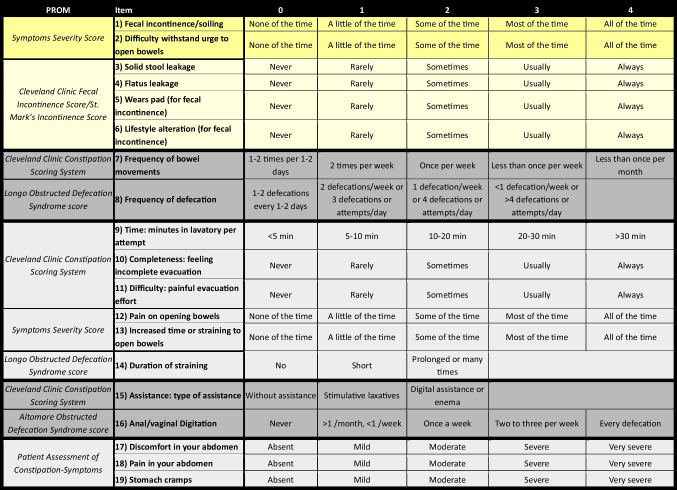
The final Defecation Disorders Scoring System was derived from the seven selected Patient-Reported Outcome Measures and divided into five components (incontinence, frequency of bowel movements and defecation, evacuation effort and duration, type of assistance, abdominal discomfort). To allow more accurate comparisons between distinct patient subgroups affected by a single type of defecation disorder, the Defecation Disorders Scoring System could be divided according to the underlying disorder (fecal incontinence domain in yellow, chronic constipation/obstructed defecation syndrome domain in grey)

**Fig. 3:**
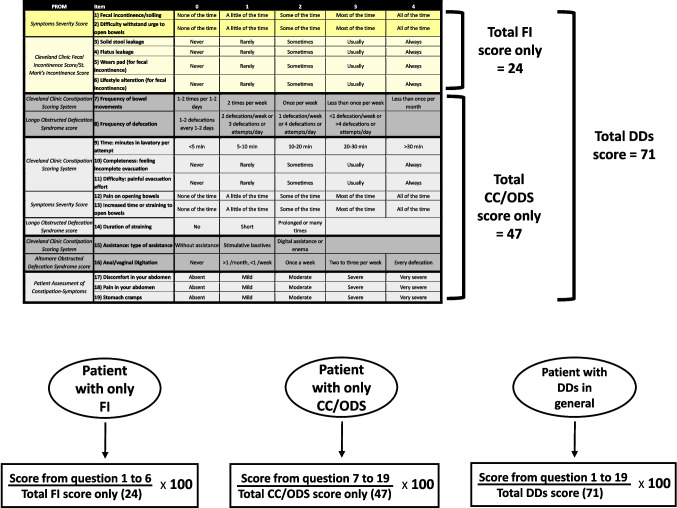
The upper part of the figure shows the Defecation Disorders Scoring System (DDSS), as previously presented in Table 2, along with the corresponding total score for patients who may be suffering from only fecal incontinence (yellow section), only chronic constipation/obstructed defecation syndrome (gray section), or defecation disorders in general. The lower part of the figure displays the algorithm for calculating the DDSS score for the specific patient subgroups (fecal incontinence only, chronic constipation/obstructed defecation syndrome only, defecation disorders in general), along with the corresponding domain-specific percentages, which could potentially allow for weighted comparisons between the different subgroups. FI, fecal incontinence; CC, chronic constipation; ODS, obstructed defecation syndrome; DDs, defecation disorders

More specifically, the overall DDSS score, obtained by summing all selected items, reached a maximum value of 71 when evaluating a patient presenting with DDs in general. However, to enable more accurate comparisons between distinct patient subgroups (suffering from FI only, CC/ODS only, or DDs in general), the DDSS could be divided according to the underlying disorder, with calculation of the corresponding percentage score for each domain (as described in Fig. 3). For instance, in patients with isolated FI, the maximum achievable score would be 24 (yellow section, Table 2), whereas in those with isolated CC/ODS, the maximum would be 47 (grey section, Table 2). Expressing each patient’s score as a percentage of the domain-specific maximum allows one to obtain a normalized value reflecting the symptom severity as perceived by the patient, acknowledging that patients with severe FI could not be directly comparable to those with severe CC/ODS, since the total possible score differs (approximately half for FI).

Finally, the internal consistency of the overall 19-item scale and each of the five components was assessed. The overall questionnaire showed acceptable internal consistency (*α* = 0.77). For each subscale, the incontinence component achieved excellent value (*α* = 0.91), while items related to evacuation effort and time demonstrated good internal consistency and consistently measured their constructs (*α* = 0.84). Internal consistency was also acceptable (*α* = 0.78) for abdominal discomfort, moderate (*α* = 0.65) for the type of assistance, and poor (*α* = 0.56) only for the component on frequency of bowel movements/defecation.

## Discussion

Historically, significant efforts have been made to develop suitable questionnaires for the assessment of DDs, typically addressing them separately. Since the introduction of the CCFIS in 1993 [17], more than 20 PROMs have been developed for FI and 23 for CC [7, 23–25]. Similarly, a recent systematic review identified 85 PROMs assessing pelvic floor symptoms in urogynecology patients, including DDs [6]. More recently, a consensus statement on PROM use in pelvic floor disorders and DDs was published by the Pelvic Floor Consortium; however, no systematic method for identifying such instruments was outlined, raising the risk of missing potentially relevant and impactful PROMs [26]. Additionally, many of these questionnaires have addressed overlapping aspects, often resulting in significant redundancy [18]. To minimize redundancy, this study gathered several widely used and validated PROMs from patients with RP and DDs and combined their items through a statistically valid method.

The main advantage of PROMs in clinical practice is their ability to identify up to half of patient’s symptoms, especially those related to FI, which might otherwise be missed or underestimated, significantly influencing treatment decisions [27–29]. However, PROMs have several limitations, including potential discrepancies between patient-reported outcomes and clinical and instrumental assessments. Neither can fully replace the others; instead, they should be used complementarily [30, 31]. Consequently, care must be taken to select relevant items, ensure content clarity, and choose an appropriate tool for the intended measurement while avoiding overloading questionnaires. Within this context, the present study aimed to propose a scoring system capable of analyzing multiple DD aspects “at the same time,” offering a comprehensive overview of the patient’s condition. Although the proposed DDSS is derived from existing PROMs, which may partially address overlapping DD domains (e.g., the SSS), its potential novelty lies not in introducing new domains, but in systematically consolidating and distilling all relevant aspects into a single, concise, non-redundant tool. At this early phase, the DDSS should be regarded as a composite scoring system derived from previously validated and widely used instruments, rather than as a fully developed “de novo” PROM, as it was derived exclusively through statistical methods without direct patient involvement. Accordingly, the DDSS should not be considered a standalone diagnostic tool, but rather a measure of patient-reported symptom severity that may help guide subsequent examinations and investigations. Furthermore, as the DDSS does not assess QoL, another important outcome in patients with DDs, it may be advisable to use it alongside a separate, validated QoL tool for a more comprehensive assessment. Finally, since the DDSS was derived solely from patients with RP, additional studies in broader and more heterogeneous populations are warranted to explore its generalizability and potential applicability to other pelvic floor disorders where different DDs coexist, such as low anterior resection syndrome.

Specific complexity concerns the differences between CC and ODS [10, 32]. In particular, the ODS remains poorly defined, and further efforts are needed. Nevertheless, a large number of PROMs are currently adopted to evaluate ODS, making selection challenging [10]. Therefore, this study focused on the most commonly used PROMs for both CC and ODS and sought to identify items of potential relevance for clinical and research purposes. Using a statistically robust method, only certain questions that stood out in frequency and/or terminology were retained in the DDSS, while others were considered potentially redundant. Among the selected items, only those addressing bowel movement frequency and defecation frequency could be considered redundant. However, even if they seem to express similar concepts from a clinical standpoint, both were retained because they may represent two distinct psychometric constructs. In patients with CC and/or ODS, bowel movements may not necessarily correspond to the mechanical expulsion of fecal material through the anus, suggesting that these items could really reflect different aspects. Notably, the component on frequency of bowel movement/defecation was the only one to show lower internal consistency, likely for the aforementioned reasons. Further targeted studies could explore this issue, for example, by using the DDSS to assess these two aspects separately, or by considering the possibility of reformulating/re-evaluating these items.

A key role of PROMs is to use the same scale across studies to enable reliable and straightforward comparisons, even if the scale itself is not perfect [6, 7]. In the systematic review evaluating 85 PROMs for pelvic floor symptoms, two PROMs were recommended for a comprehensive assessment of DDs’ related symptoms associated with pelvic floor disorders [6]. However, upon careful evaluation, they proved too long-winded to address only a few DD symptoms, especially those related to FI. Conversely, two other PROMs were identified as comprehensive questionnaires for pelvic floor disorders in the same review. However, one of these involves too many disorders, with the risk that items related to bladder, sexual, and pelvic organ prolapse dysfunctions could heavily influence the final score [33]. The second questionnaire, although described as a very comprehensive, online-friendly electronic tool for pelvic floor symptoms, is costly and no longer available [34]. The present study aimed to simplify the PROM selection by proposing a score as comprehensive as possible for DDs, without being overly lengthy or complex. Statistical analysis helped us to reduce the number of items from various scoring systems evaluating DDs. As a result, the proposed DDSS could potentially help overcome the difficulties in choosing among existing scores and contextualize different aspects of the same conditions grouped under the term “DDs.” However, as mentioned above, although the DDSS is based on already validated items, future studies are needed to assess its reliability, validity, and responsiveness.

Several PROMs for CC were developed in digital form [7]. Given the widespread use of smart devices, electronic PROMs are a viable option for remote, patient-led monitoring. In fact, consistent symptoms reported digitally could enhance patient-clinician communication, facilitate the detection of unrecognized problems, and improve patients’ health behaviors, including self-management and empowerment. Additionally, greater standardization could maximize the potential of electronic PROMs. Therefore, we would like to convert the DDSS into an online-friendly tool. Moreover, in an electronic format, the DDSS could be more easily subdivided according to the underlying disorder, allowing the calculation of domain-specific percentage scores that may help explore weighted comparisons between patient subgroups affected by different types of DDs.

A further interesting aspect emerged from the statistical comparison between validated PROMs for FI regarding the similarity between soiling and liquid stool incontinence questions. Probably, patients did not clearly distinguish between them, highlighting the need for clearer information and clarification regarding these specific differences [35]. Likewise, significant effort should be made to enable patients to discuss FI and, in general, DDs, reducing embarrassment and psychological barriers. Recently, a FI core outcome set was developed using the Delphi method to identify a minimum set of outcomes to be measured in FI studies [36]. However, the authors argued that future research is needed to find the appropriate measurement instruments for each outcome. Similarly, a systematic review on core outcome sets in patients with slow transit constipation reported excessive variability in both outcomes and measurement tools [37]. Nonetheless, a better understanding of patient and clinician priorities when facing DDs is essential for selecting the most suitable treatment, as patient-perceived severity often differs from clinician-perceived severity, with underestimation or overestimation of their bowel symptoms [38]. In this context, the DDSS may be considered a preliminary framework to support the assessment of DDs.

This study has several limitations. First, it was conducted on a relatively small sample from a single-center population. Moreover, although the DDSS could potentially be applied to other populations (e.g., non-surgical candidates, primary FI or CC without RP), in the present study, it specifically referred to patients awaiting surgery for RP, which may limit the generalizability of our findings. Similarly, as specified in the “Methods” section, only female patients were included due to the higher prevalence of RP in this population. However, DDs can also affect men, with potential differences in symptoms and clinical manifestations between sexes. Therefore, we acknowledge that future studies involving larger populations, including other types of DDs and male patients, will be required to further assess the validity and utility of the DDSS. Furthermore, future validation of the DDSS will aim to assess test–retest reliability, as well as convergent and discriminant validity relative to existing PROMs and clinical assessments, and responsiveness to change. Only seven PROMs were selected, two of which have not been formally validated (Longo ODS score and SSS). Nevertheless, although many other questionnaires could be considered, only the most widely used and scientifically recognized tools in the management of patients with DDs were chosen to facilitate the item selection process. Finally, as previously mentioned, the DDSS does not specifically assess QoL, as it focuses on symptom-related items. The only indirect measure of QoL impairment is the question about lifestyle changes related to FI symptoms, which was statistically derived from the CCFIS and SMIS. However, we believe that QoL should be evaluated separately and in greater detail to better capture both the severity of symptoms and their impact on QoL.

## Conclusion

This study proposes a scoring system specifically designed for DDs, statistically derived from existing validated PROMs, and aimed at reducing item redundancy. A final set of 19 items was identified and organized into five core components addressing incontinence, frequency of bowel movements/defecation, evacuation effort and duration, type of assistance, and abdominal discomfort. Despite some limitations, the proposed questionnaire could potentially provide a concise, comprehensive tool for assessing multiple DD aspects, potentially available in electronic format. Future studies will be required to further evaluate and validate DDSS across different patient populations.

## Data Availability

No datasets were generated or analysed during the current study.
